# Access to healthcare for deaf people: a model from a middle-income country in Latin America

**DOI:** 10.11606/s1518-8787.2020054001864

**Published:** 2020-01-23

**Authors:** Eduardo Fuentes-López, Adrian Fuente

**Affiliations:** I Pontificia Universidad Católica de ChileFacultad de MedicinaDepartamento de Ciencias de la SaludSantiagoChilePontificia Universidad Católica de Chile. Facultad de Medicina. Departamento de Ciencias de la Salud. Santiago, Chile; II Université de MontréalFaculté de médecineÉcole d’orthophonie et d’audiologieMontréalQuébecCanadaUniversité de Montréal. Faculté de médecine. École d’orthophonie et d’audiologie. Montréal, Québec, Canada; III Centre de recherchel’Institut universitaire de gériatrie de MontréalMontréalQuébecCanadaCentre de recherche de l’Institut universitaire de gériatrie de Montréal. Montréal, Québec, Canada

**Keywords:** Persons with Hearing Impairments, Effective Access to Health Services, Socioeconomic Factors, Health Status Disparities

## Abstract

**OBJECTIVE:**

To determine if there are existing healthcare access inequities among the deaf Chilean population when compared to the general Chilean population.

**METHODS:**

Data were obtained from a population-based national survey in Chile. In total, 745 prelingually deaf individuals were identified. The number of times the person used the healthcare system was dichotomized and analyzed using a multivariate logistic regression model.

**RESULTS:**

Prelingually deaf people had lower incomes, fewer years of education, and greater rates of unemployment and poverty when compared with the general population. Moreover, they visited more general practitioners, mental health specialists, and other medical specialists. On average, they attended more appointments for depression but had fewer general checkups and gynecological appointments than the general population.

**CONCLUSIONS:**

Deaf people in Chile have a lower socioeconomic status than the rest of the Chilean population. The results from this study are similar to the findings reported for high-income countries, despite differences in the magnitude of the associations between being deaf and healthcare access. Further studies should be conducted to determine the health status of deaf people in Chile and other Latin American countries and what factors are associated with a significantly lower prevalence of gynecological appointments among deaf women when compared with non-deaf women.

## INTRODUCTION

Low socioeconomic status has been associated with having a disability. In the United States, Minkler et al.^1^ observed that people living below the poverty line aged between 55 and 85 years were six times more likely to have a physical limitation. Regarding the health status of deaf people, Emond et al.^2^ reported that deaf people in the United Kingdom were more likely to suffer chronic, undiagnosed health conditions such as obesity, high blood pressure, and cardiovascular diseases than the rest of the population. In the US, McKee et al.^3^ reported that deaf users of American Sign Language (ASL) in Rochester utilized emergency services almost twice as often as the general population. This community also presents a higher prevalence of obesity, partner violence, and suicide than the general population^4^. Similarly, Genther et al.^5^ reported that adults with late-onset hearing loss accounted for a higher number of hospitalizations than the general population. Moreover, they reported poorer physical and mental health in this group.

The combination of the lack of education and high unemployment rates observed among deaf individuals has been suggested as a factor that increases their potential for mental health problems^6^. We must note that several studies have shown that the prevalence of mental problems is higher in deaf people than in the general population^7^.

Most of the studies cited above come from high-income countries. Little is known about the social and economic gradient among the deaf in low- and middle-income countries. Kuenburg et al.^11^ suggested that many deaf people from low - and middle-income countries may be suffering from much greater health disparities than what was found in their systematic review, where most of the studies reviewed came from high-income countries. We hypothesize that in countries with marked inequities, as in the case of Latin America, being deaf is a powerful stratification marker, even more important than income, education, or gender.

Given this context, the aims of this study were to determine if there is a socioeconomic gradient in prelingual deaf people and to determine if healthcare access inequities are observed in this population in Chile. This country was taken as an example of a middle-income country from Latin America. We specifically investigated the influence of deafness and other stratification markers (e.g., income level, educational level, type of health insurance system) on the access to healthcare services. The term “deaf” is considered as the definition proposed by the Canadian Association of the Deaf (CAD)^12^. The CAD defines “deaf” as a medical/audiological term referring to individuals who have little or no functional hearing. The CAD also mentions that the collective noun “the deaf” may be used to refer to people who are medically deaf but who do not necessarily identify with the Deaf Community.

## METHODS

### Study design and data source

This work was based on the 2011 Chilean National Socioeconomic Survey (NSS). This is an instrument that has been systematically applied in Chile since 1985 and is one of the country’s main ways of evaluating the impact of social policies. The survey has a cross-sectional design using a random and stratified sample based on geographical area and population size^13^. The NSS is representative of the national and regional populations (the country is divided into 15 regions). First, residence areas were established, and then households and their occupants were identified^13^.

The head of the household was interviewed to obtain data about those who live with them. These interviews were conducted in person by trained interviewers using written questionnaires and, in some cases, visual aids were used to help the person being interviewed better understand the possible answers for a particular question^14^. All respondents were informed about the study and signed an informed consent form at the beginning of each interview.

#### Participants

The 2011 NSS included lay questions to identify family members who were prelingually deaf. The interviewee (head of the household or a parent in the case of children) was asked to “mention people who are completely deaf or who have some kind of hearing problem and who, despite using hearing aids, cannot hear properly (cannot understand a conversation held at a normal volume)”. “People who can correct their condition using hearing aids” were excluded^13^. The answer to the question “What is the origin of this condition?” was also explored. Only individuals who were identified as being prelingually deaf were selected for study purposes. The objective of this question was to identify prelingually deaf individuals (i.e., people suffering from bilateral severe-to-profound hearing loss).

Furthermore, socioeconomic indicators (i.e., educational level, employment status, and income level) included in the 2011 NSS were obtained^14^. Subsequently, the responses about the frequency of use of healthcare services (i.e., appointments to general practitioners, mental health specialists, and medical specialists other than mental health) were retrieved for further analyses. These data were obtained according to variables such as age, healthcare insurance system, and whether the person was prelingually deaf or not.

The number of checkups for chronic illnesses, routine checkups for adults and older adults, gynecological checkups, Papanicolaou tests, and mammograms were also obtained. Finally, the number of bookings for appointments related to the priority health conditions that are fully covered by any health insurance system in Chile was also obtained (see Frenz et al.^15^ for a list of such health conditions).

## Statistical analyses

Initially, the proportion (i.e., prevalence) of both prelingually deaf people and the general population stratified by age (different age ranges), educational level, income level, and health insurance system was obtained. The statistical variance for the proportions was treated separately by group (deaf people versus the general population). Then, the use of the healthcare system by each possible specialty (i.e., appointments with general practitioners [GP], mental health specialists, and medical specialists other than mental health specialists) was dichotomized (i.e., no use versus one or more appointments). Proportions for both deaf people and the general population aged 21 years and older were obtained.

## Socioeconomic inequities between deaf people and general population

Multivariate logistic regressions were created to model the association between the independent variable (deaf versus non-deaf) and the dependent variable of medical appointments (none versus one or more) adjusted by the demographic factors (gender, income quintile, health insurance system, and whether or not the person has ever worked). One model was created for each of the dependent variables (i.e., appointments with GP, mental health specialists, and medical specialists other than mental health specialists). Goodness of fit was evaluated through the test proposed by Archer & Lemeshow, considering the complex design of the 2011 NSS^16^. To avoid overestimating the degree of association between variables, differences for the marginal adjusted prevalence were obtained when necessary^17^. This adjustment was based on a direct standardization of the prelingually deaf population. Statistical significance values were obtained using the Wald test.

Then, marginal logistic regressions were created to model the association between the independent variable (deaf versus non-deaf) and the dependent variables of (1) checkups for chronic illnesses, (2) routine checkups for the older adults, (3) gynecological checkups, (4) Papanicolaou tests, (5) mammograms, (6) depression, and (7) one or more of the 22 selected health conditions that have been prioritized by the Chilean Ministry of Health. Thus, seven models were created and adjusted based on the demographic factors (gender, income quintile, health insurance system, and whether the person had ever worked). The dependent variable was dichotomized as none versus one or more (e.g., gynecological checkups, Papanicolaou test). For model 2, only older adults were included, and for models 3, 4, and 5, only women were included in the analysis. From these logistic models, both prevalence ratio and prevalence difference were estimated.

For all analyses, the STATA module for complex samples (SVY command) was used, indicating that the survey was a “multiple-stage” design^18^. Sampling weights (pweights in STATA version 12), variables that identified the strata, and the primary sampling units (psu or clusters) were set. A Taylor-linearized variance estimation was used to estimate the standard error^18^. The alpha considered was 5% with a 95% confidence interval (95%CI) for all statistical tests.

## RESULTS

### Prevalence of prelingually deaf people in Chile

Based on the 2011 NSS, there are 62,008 (95% CI 52,653–71,363) people in Chile who are prelingually deaf (i.e., bilateral severe-to-profound hearing loss). This number was extrapolated from 745 people who were identified in the survey as being prelingually deaf. According to this number, the prevalence of deafness from birth is 0.37% (95%CI 0.31–0.43). The age group distribution is homogenous, except in some segments where a lower population density is observed (Table 1). The distribution by gender is similar: 50.8% males (95%CI 43.7–58.0) and 49.2% females (95%CI 42.0–56.3).

**Table 1:** Prevalence of prelingually deaf people in Chile by age according to the 2011 NSS.

Age	0 to 14	15 to 25	26 to 44	45 to 59	60 and over	Total

	(95% CI)	(95% CI)	(95% CI)	(95% CI)	(95% CI)	(95% CI)
N*	15,318 (9,889–20,747)	12,169 (8,888–15,450)	13,744 (10,191– 17,297)	8,512 (6,083–10,941)	12,265 (8,386–16,144)	62,008 (52,653–71,363)
Proportion	0.43 (0.30–0.61)	0.36 (0.27–0.47)	0.33 (0.26–0.43)	0.26 (0.20–0.35)	0.47 (0.34–0.64)	0.37 (0.31– 0.43)

* Based on the expanded sample (sampling weight).

NSS: National Socioeconomic Survey.

### Socioeconomic gradient

**Figure 1:**
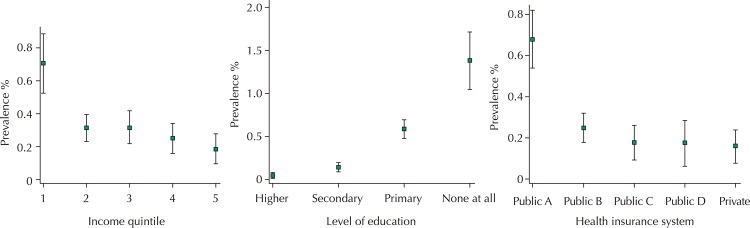
Socioeconomic gradient for the prevalence of deaf people. Left panel: income quintiles from the poorest (1st) quintile to the richest (5th) quintile. Middle panel: level of education. Right panel: health insurance system. The public health system comprises four categories depending on the user’s income levels, ranging from “A” for people with the lowest income level to “D” for people with the highest income level. Results represent 95% confidence intervals.

A socioeconomic gradient was observed in the prevalence of prelingually deaf people. This was observed to a greater extent in the quintile with the lowest income (0.7% in the 1st quintile; 95%CI 0.5–0.9), and especially in the segment with no formal education (1.38%; 95%CI 1.0–1.7). The percentage of prelingually deaf people covered by the public health system in the “A” section was 0.7% (95%CI 0.5–0.8), including homeless people, those with no financial resources, and individuals who receive basic pensions or family subsidies (Figure 1).

#### Differences between deaf people and the general population for income, education, and employment

Prelingually deaf people aged 21 years and older were concentrated in the first (lowest income) quintile at 38.3% (95%CI 31.5–45.2), whereas 8.4% of prelingually deaf people (95%CI 3.5–13.3) belonged to the richest quintile. We must note that around 60% of prelingually deaf people were found in the two poorest quintiles. Regarding the general population aged 21 years and older, 18.9% (95%CI 17.9–20.0) belonged to the richest quintile, and 18.4% (95%CI 17.6–19.2) to the poorest quintile.

Regarding the educational level, 21.7% (95%CI 17.3–26.9) of prelingually deaf people indicated that they had never attended any kind of school. A similar percentage (24.1%, 95%CI 18.7–30.4) mentioned that they had received special education. In total, 20.6% (95%CI 15.6–25.4) indicated that they received primary education, 11.2% (95%CI 7.5–16.4) received secondary education, and 2.8% (95%CI 1.5–5.4) had attended a technical-vocational school. Only 2.0% (95%CI 0.9–3.0) mentioned that they had been to or were currently attending university. Finally, 64.5% (95%CI 56.7–71.6) of deaf people indicated that they knew how to read and write.

Regarding the general population aged 21 years and older, 2.8% (95%CI 2.6–3.0) never attended any kind of school and 0.3% (95%CI 0.2–0.4) attended a special education school. In total, 17.3% (16.3–18.3) reported tertiary studies, and 96.3% (95%CI 96.1–96.5) indicated that they knew how to read and write.

Regarding the history of employment, only 39.1% (95%CI 30.18–48.10) of prelingually deaf people had worked at any given time. This number was significantly lower than the 66% observed in the general population (95%CI 64.43–67.47), and 70% (95%CI 62.3–77.8) of prelingually deaf people mentioned that they would not be able to work even if they were offered a job. In addition, 95% (95%CI 92.3–99.3) of prelingually deaf people were not looking for work or were not pursuing self-employment. When asked why they were not looking for work, 56.8% (95%CI 47.9–65.6) responded that it was because they had a disability.

In total, 94.1% (95%CI 90.8–97.5) of prelingually deaf people were part of the public healthcare system, and only 3.5% (95%CI 0.5–6.6) had private health insurance. The remaining participants were covered by a different system (0.9%; 95%CI 0.2–1.6), such as the one provided by the armed forces, or did not have any health insurance (0.14%; 95%CI 0.02–0.27).

Regarding medical appointments, prelingually deaf people as a group had attended a higher number of GP, mental health, and other medical specialist appointments when compared to the general population. In total, 10.8% (95%CI 6.3–15.2) of prelingually deaf people aged 21 years and older had attended at least one mental health appointment when compared to 2.7% (95%CI 2.5–2.9) of the general population. Among prelingually deaf people who had attended at least one appointment for mental health, it was observed that they were mainly (a) aged between 21 and 30 years (14.0%, 95%CI 2.2–25.7), (b) female (12.2%, 95%CI 5.7–18.7), (c) unemployed and had never worked in their lives (14.6%, 95%CI 7.1–22.1), and either (d) had attended primary school only (13.6%; 95%CI 6.2–21.0) or (e) had no formal education at all (9.5%; 95%CI 2.5–16.4). Details are shown in Table 2.

**Table 2:** Proportion of GP, mental health and medical specialist other than mental health specialist appointments according to the 2011 NSS stratified by some socioeconomic indicators in people aged 21 years and older.*

	Appointment with GP (95%CI)		Appointment with mental health specialist (95%CI)		Appointment with medical specialist other than mental health specialist (95%CI)	
		
	General population	Deaf population	General population	Deaf population	General population	Deaf population
Age						
21–30 years	10.6 (9.7–11.5)	26.9 (13.8–40.0)	2.0 (1.7–2.3)	14.0 (2.2–25.7)	7.3 (6.6–8.1)	26.7 (12.0–41.4)
31–44 years	13.6 (12.6–14.6)	32.7 (17.9–47.4)	2.8 (2.4–3.2)	12.7 (3.7–21.6)	8.8 (8.2–9.6)	14.5 (4.8–24.2)
45–59 years	18.3 (17.4–19.3)	27.0 (14.5–39.5)	3.1 (2.6–3.6)	7.9 (1.0–14.8)	11.9 (11.2–12.7)	20.1 (6.7–33.6)
60 and over	28.9 (27.4–30.5)	43.7 (28.3–59.1)	2.9 (2.5–3.4)	8.7 (1.8–15.4)	18.1 (17.0–19.2)	25.7 (11.0–40.4)
Gender						
Female	21.0 (20.2–21.8)	40.3 (28.1–52.5)	3.7 (0.2–3.4)	12.2 (5.7–18.7)	13.7 (13.0–14.3)	25.4 (14.7–36.1)
Male	13.5 (12.8–14.1)	25.6 (16.9–34.2)	1.5 (0.1–1.3)	9.1 (2.8–15.3)	8.7 (8.0–9.3)	18.4 (9.3–27.5)
Income quintile						
5th quintile	19.1 (17.2–19.8)	42.3 (10.6–73.9)	2.7 (2.2–3.1)	10.9 (0.0–26.9)	18.2 (16.8–19.6)	53.0 (23.6–82.4)
4th quintile	16.8 (15.5–17.5)	27.8 (10.3–45.3)	2.2 (1.8–2.6)	7.1 (0.0–16.0)	11.2 (10.3–12.0)	15.7 (19.4–29.5)
3rd quintile	16.9 (15.5–18.3)	51.7 (32.7–70.7)	2.7 (2.2–3.3)	13.0 (1.3–24.3)	10.1 (9.3–11.0)	30.5 (11.2–50.0)
2nd quintile	16.5 (15.7–17.9)	29.5 (17.2–41.8)	2.9 (2.4–3.3)	10.0 (1.8–18.2)	9.5 (8.6–10.3)	21.8 (9.6–34.1)
1st quintile	18.6 (17.7–20.6)	26.1 (15.4–36.8)	3.1 (2.7–3.5)	11.5 (3.8–19.2)	8.2 (7.5–8.9)	13.8 (6.1–21.5)
Whether the person has ever worked						
Yes	22.5 (21.5–23.5)	41.9 (26.2–57.5)	3.8 (3.3–4.2)	8.8 (1.8–15.7)	14.6 (13.8–15.3)	24.9 (10.1–39.6)
No	20.1 (18.5–21.7)	25.8 (16.6–34.9)	3.4 (2.6–4.3)	14.6 (7.1–22.1)	12.1 (10.8–13.5)	18.9 (10.3–27.4)
Educational level						
Higher	15.8 (14.7–16.9)	52.6 (27.4–77.9)	2.7 (2.3–3.1)	7.9 (0.0–20.2)	14.3 (13.2–15.4)	36.8 (11.5–62.1)
Secondary	15.7 (14.9–16.6)	36.3 (20.7 -52.0)	2.6 (2.4–2.9)	5.6 (0.0–13.0)	10.3 (9.5–11.0)	17.3 (5.4–29.4)
Primary	21.1 (20.2–22.0)	36.4 (24.4–48.5)	2.9 (2.5–3.4)	13.6 (6.2–21.0)	10.5 (9.9–11.2)	31.8 (19.3–44.2)
none at all	26.4 (22.7–30.2)	23.5 (11.5–35.6)	2.3 (1.6 –3.1)	9.5 (2.5–16.4)	9.6 (8.2–10.9)	6.3 (2.3–10.2)
Health insurance system						
Public	18.0 (17.3–18.7)	33.4 (24.9–42.0)	2.8 (2.5–3.0)	11.3 (6.5–16.1)	10.3 (9.8–10.8)	19.6 (12.3–26.9)
Private	17.7 (16.1–19.3)	21.6 (0.0–48.1)	2.9 (2.4–3.4)	4.9 (0.0–15.0)	19.5 (18.1–21.0)	69.3 (37.0–100.0)
Other	11.1 (8.5–13.6)	29.0 (0.0–65.0)	1.4 (0.7–2.1)	10.6 (0.0–30.9)	5.8 (4.1–7.5)	20.8 (0.0–43.8)
None	6.1 (5.0–7.3)	(no observations)	1.4 (0.8 –2.0)	(no observations)	4.5 (3.5–5.4)	(no observations)

* Based on the expanded sample (sampling weight).

## Inequity in the access to healthcare services

A higher adjusted odds ratio (OR) was observed in prelingually deaf people when compared to the general population for GP, mental health, and other medical specialist appointments (Table 3). Although expected differences in size were found, the results were the same when estimating the adjusted marginal prevalence using the characteristics of the prelingually deaf population (Table 4). The educational level was found to be associated with medical specialist and mental health appointments in the population aged 21 years and older (Table 3).

**Table 3:** Adjusted odds ratios for the deaf population aged 21 years and older, as compared to the general population for GP, mental health and medical specialist other than mental health specialist appointments.

	GP OR (95%CI)	Mental health OR (95%CI)	Specialist other than mental health specialist OR (95%CI)
Deafness‡, ‡‡	1.78 (1.18–2.66)**	4.72 (3.01–8.24)***	2.50 (1.49–4.19)***
Goodness of fit *p* value	0.465	0.556	0.129

‡ Adjusted for age (21 years and older), gender, income quintile, whether the person has ever worked, level of education, and health insurance system.

‡‡ Stratified analysis in deaf population aged 21 years and older.

**p < 0.01, *** p < 0.001

**Table 4:** Prevalence, prevalence ratio and prevalence difference for non-access to certain healthcare services relating to women’s health.

	General population (95% CI)	Deaf people (95% CI)	Prevalence ratio (95% CI)	Prevalence difference (95% CI)
No gynecological checkup ‡	83.8 (81.8–85.8)	97.4 (93.5–100.0)	1.2 (1.1–1.2)***	13.6 (9.2–18.0)***
No Papanicolaou test ‡ ‡	41.8 (40.2–43.4)	65.3 (56.1–74.5)	1.6 (1.3–1.8)***	23.5 (14.1–32.9)***
No mammogram test §	37.2 (35.4–39.0)	42.6 (25.7–59.5)	1.2 (0.7–1.6)	5.4 (-0.1–22.6)

*** p < 0.001

‡ The variable was reversed and recodified: no gynecological checkup in the last 3 years (women only).

‡ ‡ The variable was reversed and recodified: no Papanicolaou test in the last 3 years (women only).

§ The variable was reversed and recodified: no mammogram test in the last 3 years (women only).

### Medical appointments for routine checkups

Prelingually deaf people were more likely to have at least one routine checkup (OR: 2.6; 95%CI 1.53–3.05) than the general population. This probability increased with age (eight times higher in people aged 60 years and over) and among those in the lowest income quintile (OR: 1.18; 95%CI 1.03–1.35). This is consistent with the higher probability of older adults having had a routine checkup in their lifetime (OR: 2.39; 95%CI 1.01–5.66). Despite this, prelingually deaf women were 88% less likely to have had a gynecological checkup in their lifetime, and 65.7% less likely to have had a Papanicolaou test (OR: 0.34; 95%CI 0.22–0.54) than the general population. Even when estimating the marginal adjusted prevalence for the prelingually deaf population, the differences between the deaf and the general population for medical appointments for routine checkups were maintained (Table 4).

### Treatment for health conditions

In the group aged 21 years and older, the likelihood of being in treatment for depression was 2.5 times higher (95%CI 1.14–5.50; p < 0.05) among the prelingually deaf population than it was among the general population. Moreover, the probability of prelingually deaf people being in treatment for other priority health conditions was 4.11 times higher (95%CI 2.68–6.30; p < 0.01) than it was for the general population.

## DISCUSSION

### Socioeconomic gradient

Results from this study show that prelingually deaf people in Chile had a significantly lower level of education and income when compared to the general population. Similar results have been observed in the United States by Blanchfield et al.^19^ In Australia, people with severe to profound hearing loss are more likely to have a low educational level and low income and to be unemployed when compared to the general population^20^. No reports from middle-income countries have been found in the existing literature.

Another significant result is the 56.8% of prelingually deaf people who reported that they had never looked for a job due to their disability. These results are similar to those found in the Australian population, where 86% indicated that hearing loss limited their chances of employment^20^. In Canada, 19% of young prelingually deaf people perceived themselves as “victims of discrimination” when looking for a job. This percentage was much higher than the percentage of young people with other communication disabilities who reported feeling discriminated against because of their disability (8.8%)^21^. No reports from middle-income economies were found in the existing literature.

The percentage of deaf people who had access to university education in this study (2%) was similar to the 5% reported by Blanchfield et al.^19^ in the United States. Furthermore, the higher prevalence of prelingually deaf people in the lowest income quintiles corroborates the results found in a systematic review about societal-level risk factors associated with pediatric hearing loss^22^.

### Health-related inequities

We observed that deaf people were more likely to visit a GP or a medical specialist than the general population. They were also more likely to be in treatment for depression and other priority health conditions. However, in the case of deaf women, they were less likely to undertake routine examinations related to women’s health. Differences between the deaf and the general population were controlled for some demographic variables such as gender, age, educational level, and income level. Therefore, these results indicate that, when compared to the general population, deaf people in Chile do not have reduced access to healthcare services, except for gynecology. However, these results may indicate that their health status is poorer than the health of the general population. For example, deaf people aged 21 years and older were 2.5 times more likely to be in treatment for depression than the general population. Therefore, being deaf in Chile is significantly associated with a higher prevalence of mental problems. Despite the similarities in results between this study and reports from studies conducted in high-income countries regarding the association between being prelingually deaf and the number of appointments with medical specialists, differences in the magnitude of such associations are observed when prelingually deaf people and non-deaf people are compared. For example, the OR for Canadian adults consulting with a professional for depression, as reported by Woodcock & Pole^23^, was 1.59, compared to the 2.5 OR found in this study.

As mentioned above, despite deaf people having more medical appointments and being more frequently in treatment for several priority health conditions, deaf women were significantly less likely to attend a gynecological examination. Woodcock & Pole^23^ found a 1.52 OR for attending an appointment with a GP among deaf people when compared to the general population, and no significant association between being prelingually deaf and having a Papanicolaou test was found. We found a 1.78 OR for attending an appointment with the GP, and prelingually deaf women were significantly less likely to have a Papanicolaou test. We must mention that Woodcock & Pole^23^ did not adjust for income levels, educational level, and type of health insurance, whereas the ORs were adjusted for these variables in the present study. Thus, differences in ORs between both studies may be even larger.

Therefore, the question that remains to be answered is why deaf women have less access to gynecological services when deaf people as a group show significantly higher access to other health services. We hypothesize that an interaction among different factors may explain such result. First, a communication barrier between health staff and users may lead to deaf women feeling that they are in a much lower position when compared to the healthcare staff. Second, healthcare staff in Chile may not be well prepared to work with deaf people since they generally do not know sign language. Therefore, deaf women may feel embarrassed in a situation where healthcare professional with whom they cannot properly communicate are checking their genitals.

Finally, societal aspects may play an important role. The way that Chilean society is organized, along with its cultural values, may lead a large portion of society to view prelingually deaf people experiencing inequity as something normal. Definitive signs of this exclusion are found in the country’s legislation. Although prelingually deaf people do not necessarily have trouble speaking, they are often considered unintelligent regardless of their actual aptitude for communication. Furthermore, they are considered by the civil and penal codes as being on the same level as people with cognitive difficulties (e.g., dementia). According to Herrera^24^, prelingually deaf people in Chile have been excluded from making decisions about problems that affect them directly, such as education, health, and employment, reflecting their invisibility in society. This may be the case for gynecological exams as well.

In summary, deaf people were significantly more likely to attend healthcare services than the general population. Therefore, we hypothesize that deaf people’s health status is poorer than that of the general population. Since this aspect was not investigated in this study, further research should be conducted to investigate health outcomes in Chilean deaf people. Another major finding in this study relates to deaf women. They were significantly less likely to have a gynecological examination than their non-deaf peers. Further studies should investigate the factors that are associated with this finding, and future campaigns regarding women’s health in the Chilean deaf community may be put forward.

### Limitations of the study

We must stress that study data were obtained from the head of the household, most likely a parent in the case of those aged 20 years and below. This type of methodology for data collection may be subjected to bias because it relies on memory. Moreover, not all the questions may be understood, and some answers could be imprecise, given that this person has not lived through the experience they are referring to. Furthermore, if the deaf person was interviewed, communication barriers with the interviewer may have occurred. All this may have happened even if the interviewers were trained to provide visual aids when needed.

Another aspect to be considered is that in this type of survey, to specify an exact level of disability is not easy. Despite these limitations, the prevalence of deafness is very similar to the global estimations of severe-to-profound hearing loss given by the WHO in 2011 (0.5%, fluctuating between 0.3 and 0.8%)^25^. This may indicate that this type of condition is easily identified, given the variety of communication difficulties it causes. Ferrite et al.^26^ found that self-reported hearing loss has adequate validity for the detection of moderate and severe hearing losses. Furthermore, this study did not investigate deafness other than that with an early onset in life (prelingual), as well as other forms of hearing loss that may affect communication.

Finally, this study investigated whether deaf people could or could not access certain healthcare services. The ease of access for and quality of such services provided to deaf people when compared to the general population were not investigated. Similarly, the general health status of deaf people in Chile when compared to the general population was not explored in this study. Further studies addressing these aspects should be conducted.

## CONCLUSION

This study showed that prelingually deaf people in Chile had lower incomes and lower levels of education, experiencing more unemployment and poverty than non-deaf people. Moreover, deaf people were significantly more likely to be treated for depression and as a group were more likely to have visited a GP and medical specialists other than mental health specialists. This suggests that deaf people in Chile may have poorer health status than the general population. However, communication barriers may explain, at least in part, the differences between the deaf and the general population for medical appointments. Despite these results, deaf women were significantly less likely to have visited a gynecologist or to have undergone a Papanicolaou test or a mammogram. Similarly, among older adults, the deaf were less likely to have undergone routine checkups for chronic health conditions. We hypothesize that communication barriers may be the cause of such differences. The results from this study are similar to the findings reported from high-income countries, despite differences in the magnitude of the associations between prelingually deaf people and healthcare access.
